# DOWNWARD DEMENTIA TREND GOES INTO REVERSE IN ENGLAND AND WALES? ENGLISH LONGITUDINAL STUDY OF AGING 2002–2019

**DOI:** 10.1093/geroni/igad104.0646

**Published:** 2023-12-21

**Authors:** Yuntao Chen, Piotr Bandosz, George Stoye, Yuyang Liu, Sophia Lobanov-Rostovsky, Eric French, Jing Liao, Eric Brunner

**Affiliations:** University College London, London, England, United Kingdom; Division of Prevention Medicine & Education, Medical University of Gdansk, Gdansk, Pomorskie, Poland; Institute for Fiscal Studies, London, England, United Kingdom; Sun Yat-sen University, Guangzhou, Guangdong, China (People’s Republic); University College London, London, England, United Kingdom; Faculty of Economics, University of Cambridge, Cambridge, England, United Kingdom; Sun Yat-sen University, Guangzhou, Guangdong, China (People’s Republic); University College London, London, England, United Kingdom

## Abstract

Recent evidence suggests dementia incidence rates are declining in high-income countries. However, data for the trend after 2010 are scarce. We examined the temporal trend in England and Wales from 2002-2019, considering bias and non-linearity. We used population-based panel data linked to the mortality register across wave 1 (2002-2003) to wave 9 (2018-2019) of the English Longitudinal Study of Ageing. Uniform standard criteria based on cognitive and functional impairment were used to ascertain incident dementia cases. Crude incidence rates were determined in seven overlapping initially dementia-free sub-cohorts followed over four years. We estimated the age- and sex-adjusted trend of dementia incidence with Cox and multi-state models. Restricted cubic splines allowed for potential non-linearity. 19 806 people were included in the study. Crude dementia incidence declined from 2002 to 2008 (8.7 to 7.4 per 1000 person-years), and increased from 2008 to 2019 (7.4 to 10.3 per 1000 person-years). Adjusting for age and sex, and accounting for missing dementia cases due to death, estimated dementia incidence declined by 28.8% from 2002 to 2008 (incidence rate ratio 0.71, 95% CI 0.58-0.88), and increased by 25.2% from 2008 to 2016 (incidence rate ratio 1.25, 1.03-1.54). The higher education group had a sharper decline of dementia incidence from 2002 to 2008, and a smaller increase after 2008. Dementia incidence may not be declining. There was a rebound after 2008 in England and Wales. If the upward dementia incidence trend continues, along with population ageing, the burden on health and social care may be large.

